# Pharmacokinetics and residue depletion of enrofloxacin and its metabolite ciprofloxacin in land snails *(Cornu aspersum maxima)*

**DOI:** 10.2478/jvetres-2026-0024

**Published:** 2026-04-27

**Authors:** Beata Łebkowska-Wieruszewska, Monika Ziomek, Anna Gajda, Ewelina Nowacka-Kozak, Amnart Poapolathep, Mario Giorgi

**Affiliations:** 1 Department of Department of Pharmacology, Toxicology and Environmental Protection, 20-950 Lublin, Poland; 2 Department of Food Hygiene of Animal Origin, University of Life Sciences in Lublin, 20-950, Lublinw, Poland; 3 Department of Chemical Research of Food and Feed, National Veterinary Institute-National Research Institute, 24-100 Puławy, Poland; 4 Department of Pharmacology, Faculty of Veterinary Medicine, Kasetsart University, 10-900 Bangkok, Thailand 5Department of Veterinary Sciences, University of Pisa, 10-900, Bangkok, Thailand; 5 Department of Veterinary Sciences, University of Pisa, 56-122 Pisa,Italy

**Keywords:** ciprofloxacin, edible snails, enrofloxacin, haemolymph, mucus

## Abstract

**Introduction:**

The pharmacokinetics (PK) and tissue distribution of enrofloxacin (EF) and its main metabolite, ciprofloxacin (CP), were determined in land snails (*Cornu aspersum maxima*) dosed singly and multiply orally or intrahaemolymphatically (IHL).

**Material and Methods:**

In the single-dose regimen, snails received EF *via* IHL injection (1 mg/kg), gavage (30 mg/kg) or medicated wafer (30 mg/kg). Haemolymph was sampled up to 72 h post administration. In the multiple-dose regimen, snails were treated with 10 mg/kg EF *via* gavage for five days. Tissues and body fluids were sampled up to 240 h. Ultra-high-performance liquid chromatography–tandem mass spectrometry simultaneously determined the concentrations of EF and CP.

**Results:**

Concentrations of EF in haemolymph after IHL administration remained above the limit of quantification up to 72 h post dose. Enrofloxacin had a 15–19 h elimination half-life. It was more bioavailable from medicated wafers (74.6%) than from gavage (27.7%); however, variability and small sample sizes prevented statistical analysis. Concentrations of CP were inconsistently detectable and often below quantification limits, suggesting limited EF biotransformation. After multiple dosing, EF accumulated predominantly in the hepatopancreas, and less in muscle, the albumen gland and mucus. Tissue contained detectable CP but haemolymph did not. Persistence of EF was noted in tissues beyond 240 h at concentrations often exceeding the 100–300 μg/kg maximum residue limits for other food-producing species.

**Conclusion:**

This preliminary study indicates that EF clears slowly and metabolises incompletely to CP in snails, resembling its PK patterns in aquatic and reptilian species. The persistence of EF in edible tissues suggests that withdrawal periods may need to be considerably longer than those for other terrestrial animals. Further depletion studies with extended sampling times, assessment of minimum inhibitory concentration values for snail pathogens and protein binding data are warranted to support safe and rational EF use in heliciculture.

## Introduction

The demand for edible snails grows steadily, with global consumption amounting to approximately 30,000 tonnes per year (51), and forecasts predict a fivefold increase over the next 20 years. In the Atlantic and Mediterranean regions of Europe, *Cornu aspersum* (Müller, 1774), commonly known as the brown garden snail, and the Roman snail (*Helix pomatia Linnaeus*, 1758) are among the most commonly eaten species. In addition, several species of land snails, although less common in European cuisine, are consumed in other parts of the world. These include *Archachatina marginata, Achatina achatina, Achatina fulica* and *Helix lucorum* (26, 60). Snail meat is a low-energy food: 100 g of Roman snail muscle tissue provides 217 kJ of energy (51). Its primary energy-yielding components are fat (0.5–1.2%) and proteins (12–18%), as carbohydrates are minimal (41, 45). Snail meat is nutritionally valuable because it contains essential amino acids, of which Roman snails provide 4 to 5 mg per 100 g (9). The fat in snail meat is rich in unsaturated fatty acids (60 to 80%) (9, 41). Additionally, snail meat is rich in A- and B-group vitamins, including B1, and contains significant levels of sodium, potassium and iron (9).

An exclusive product from snails is white caviar, also known as escargot pearls. It is a delicacy made from the eggs of snails, and is highly regarded in Europe and globally. Its white colour contrasts with the black or red of caviar, and its earthy and nutty taste does not emulate the fish flavour of traditional caviar (33, 39). Snail eggs contain 81.41% water, 4.23% proteins, 0.09% fats, 6.62% carbohydrates and 7.65% ash, resulting in a low energetic value of 184.89 kJ (39). Despite its low nutritional value, the limited production and high cost position this food as a luxury product. The eggs are formed in the albumen gland, which supplies reserve substances and creates the egg’s outer layer during laying (1, 42). This gland can accumulate contaminants, including xenobiotics, which may transfer to the snail caviar.

In addition to their culinary value, snails also provide mucus, which is used in the cosmetic and pharmaceutical industries. Snail mucus is rich in bioactive compounds, including allantoin, collagen, elastin, glycolic acid, peptides, proteins and vitamins A, C and E. It contains antioxidants, essential metal ions (copper, iron and zinc) and antimicrobial peptides (12, 14, 28, 57). Mucin, a key component, has antibacterial, antifungal and antiviral properties, acting against *Pseudomonas aeruginosa, Staphylococcus aureus* and *Escherichia coli* (18, 43). It also supports skin regeneration, aiding in cell repair, wound healing and reduction of imperfections and discoloration (31, 52, 55). Its beneficial composition makes it a valuable resource for the cosmetic and pharmaceutical industries (35). However, because of its complexity, mucus cannot be synthetically reproduced. Since 2006, following the registration of the standardised filtrate of snail mucus in the European Commission cosmetic ingredient database, companies have been incorporating this raw material into their products (17).

Despite the antibacterial properties of mucus, snails are susceptible to various bacterial, protozoal and fungal infections (13, 20, 44). Heliciculture, characterised by a high density of individuals, can set up the conditions for potential snail infection, and this can lead to a high mortality rate. The greatest threat comes from bacteria of the *Salmonella* (20, 51), *Listeria* (19) and *Aeromonas* (50) genera, as well as *Pseudomonas* (4, 32) and *Citrobacter* spp. (48, 51).

The literature mentions the prophylactic and therapeutic use of disinfectants, such as ethacridine lactate 0.1% (59). However, there are no official reports on the off-label use of antibacterials in heliciculture, including pharmacokinetic data or information about drug residues in snail tissues or products. From discussions with snail producers, it emerged that most of them extrapolate antibacterial doses from quantities specified for other species using a trial-and-error approach. With these practices, breeders often disregard the risks of underdosing, which can lead to antibacterial resistance, or of overdosing, which may result in adverse effects and increased mortality. Breeders most commonly administer antibacterials through spraying, drinking water or mixed with feed. Little attention is paid to potential residues, as there are no regulations regarding invertebrates or the use of medications in them.

Enrofloxacin (EF) is a veterinary antibiotic in the fluoroquinolone class (8, 49). According to some breeders, it is one of the antimicrobials most used off-label in heliciculture because of its broad spectrum of activity against Gram-negative bacteria, some Gram-positive bacteria and *Mycoplasma* spp. In both vertebrate and invertebrate species, EF undergoes metabolism to form ciprofloxacin (CP), a potent antimicrobial agent widely employed in human medicine (7, 37, 53). The pharmacokinetic parameters of EF have been determined in several aquatic invertebrates (10, 23, 27, 37, 46, 58), but no information has been reported in snails so far. Therefore, the first aim of this study was to preliminarily assess the basic pharmacokinetic parameters of EF and its metabolite CP after intrahaemolymphatic and oral administration. The second aim was to evaluate their concentrations in snail tissues potentially intended for consumption (foot muscle and hepatopancreas); in tissue associated with food products, namely the albumen gland; and in products for human use, *i.e*. mucus as a cosmetic and pharmaceutical product ingredient.

## Material and Methods

### Chemicals and reagents

Pure powder analytical standards of EF and CP were purchased from Dr. Ehrenstorfer (Augsburg, Germany). The octadeuterated CP (CP-d8) used as an internal standard (IS) and heptafluorobutyric acid were from Sigma-Aldrich (St. Louis, MO, USA). Acetonitrile was obtained from J.T. Baker (Phillipsburg, NJ, USA). Formic acid (HCOOH) was from Fluka (Buchs, Switzerland). All reagents were liquid chromatography and mass spectrometry grade. Syringe filters with 0.22-μm pore size and hydrophilic polyvinylidene fluoride (PVDF) membranes were purchased from Restek (Bellefonte, PA, USA). Ultra-pure water was generated by a Millipore Milli-Q System (Millipore, Guyancourt, France).

### Animal treatment

For this study, 244 healthy adult snails (*Cornu aspersum maxima*) were supplied by a local farm (Snail breeding and sales – Aspersa Snails International, Grodziec, Poland). The animals were approximately one year old, and their body weight ranged between 20 and 30 g. The snails were determined to be clinically healthy based on initial health assessment (confirming correct movement, food intake, dry skin coat and willingness to mate) performed prior to the study by the supervising veterinarians. The snails were monitored daily by designated veterinarians who observed their behaviour and appetite.

To acclimate the snails to the study environment, they were kept in 60 × 40 cm plastic containers with lids with holes for air flow. The boxes were cleaned daily with fresh water. The temperature was maintained at 20°C and humidity at 90% in accordance with the most popular snail breeding requirements (50). The density of snails per container ranged from 20 to 25 individuals. Animals were fed with a drug-free diet prepared for farmed snails (LIRA Feed Factory, Krzywiń, Poland), twice a day. The feed contained 16.5% crude protein, 3% crude fibre, 4.5% crude fat, 29.5% crude ash, 12.5% limestone powder, 0.2% sodium, 0.75% phosphorus and 2.5% amino acid supplement. The water was supplied *ad libitum*, and the snails were sprayed with distilled water twice a day.

**Fig. 1.**
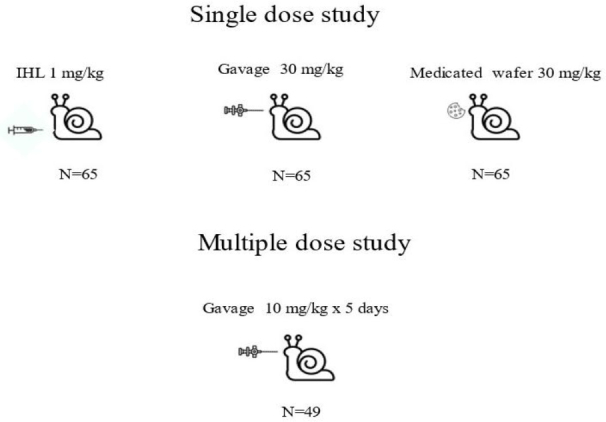
Experimental setup and regimens for administration of enrofloxacin to year-old *Cornu aspersum maxima* snails. IHL – intrahaemolymphatic

An identity code was applied to each snail shell for easier identification. The study was designed with two parallel treatment regimens (Fig. 1). The first was a single-dose treatment injected intrahaemolymphatically (IHL) at 1 mg/kg (n = 65) or given by two oral methods (*per os* – PO), namely as gavage (n = 65) and as a medicated wafer (n = 65), both at 30 mg/kg. The second was a multiple-dose treatment (PO, n = 49) at 10 mg/kg/day administered by gavage over five days. Enrofloxacin (Enflocyna Inj., 50 mg/mL; Biowet Puławy, Puławy, Poland) was diluted in saline and administered IHL into the main lymphatic vessel located topographically (based on anatomical position) using a sterile 27G 0.4 × 13 mm needle. For the PO route of administration, Enflocyna Sol. (50 mg/mL; Biowet Puławy) was diluted in saline and administered in two ways. The first was by gavage through a paediatric cannula (24G 0.7 × 19 mm) as a feeding tube, administering the whole dose directly into the crop, and the second was in a 5-mm-diameter wafer onto which a 0.2 mg/mL dose of 20 to 30 μL volume was loaded with a micropipette (simulating normal eating in field conditions). It was then checked whether the snail had swallowed the entire drug wafer, which was the starting point for time measurement for haemolymph collection. The volume of the drug administered in all stages was tailored to the body weight of each snail. A 10% error was assumed owing to the very small volumes.

Haemolymph samples of approximately 0.7 mL were collected from the main lymphatic vessel (located topographically) using a syringe with a 22G 0.7 × 40 mm needle and transferred to a 1.5 mL sterile Eppendorf tube. Haemolymph was collected from each individual only once during the entire study. It was collected at 0, 0.25 (for IHL administration only), 0.5, 0.75, 1, 1.5, 2, 4, 6, 8, 10, 24, 48 and 72 h (for PO administration only) after the single EF administration in the first treatment regimen.

During the second treatment regimen, haemolymph samples were taken every 24 h, just before each daily administration. On the fifth day, haemolymph, tissues and mucus were collected immediately before the administration of the daily dose and on the first (24 h), second (48 h), third (72 h), fourth (96 h), fifth (120 h), seventh (168 h) and ninth (216 h) day after the last treatment. The tissues were collected by section after the snail was anaesthetised with chloroform and then decapitated. An 8 g mass of muscle, 3 g of the hepatopancreas and 3 g of the albumen gland were isolated. The mucus was obtained from the pedal glands by rubbing them with a swab soaked in 3% sodium chloride (NaCl). This compound irritates the pedal epithelium and stimulates the production of mucus (52). The process was continued until the appropriate amount of approximately 1 g was obtained. The collected haemolymph, tissues and mucus were stored at a temperature of −80°C. The analysis of the stored biological matrices was carried out within 40 days from the time of collection, after each treatment regimen.

### Sample preparation for quantification of EF and CP in haemolymph, mucus, snail tissues and organs

For the clean-up of EF and CP in snail haemolymph samples, an aliquot of 100 μL of haemolymph and 100 μL of 0.1% HCOOH were placed into a polypropylene centrifuge tube. A 50 μL of IS solution (2 μg/mL) was then added and the tube contents were briefly vortexed. Next, 750 μL of water was added, and samples were vortexed and centrifuged for 3 min at 9,447 × *g*. Before UHPLC-MS/MS analysis, 500 μL of the sample was filtered into a glass vial through a 0.22 μm PVDF filter. For the EF and CP analysis in the mucus, albumen gland, muscle and hepatopancreas, a 100 mg sample of each tested material was placed into individual clean centrifuge tubes. Next, 500 μL of 0.1% HCOOH and 50 μL of IS solution (2 μg/mL) were added and mixed. Then, 350 μL of water was added, and samples were vortexed and centrifuged for 3 min at 9447 × *g*. Finally, 500 μL of the sample was filtered into a glass vial through a 0.22 mm PVDF filter before ultra-high-performance liquid chromatography with tandem mass spectrometry (UHPLC-MS/MS) analysis.

### UHPLC-MS/MS conditions

The liquid chromatography separation was performed on a Shimadzu Nexera X2 UHPLC system (Shimadzu, Kyoto, Japan) using a 50 mm × 2.1 mm × 1.8 μm Agilent Zorbax SB-C18 column (Agilent Technologies, Santa Clara, CA, USA) with the following mobile phase: 0.025% heptafluorobutyric acid in water (solvent A) and acetonitrile (solvent B). The gradient mode was as follows: 0–4.0 min 90% of solvent A, 4.01–5.30 min reduction to 20% of solvent A, and 5.31–7.0 min back to 90% of solvent A. A flow rate of 0.6 mL/min was established with an injection volume of 5 μL. The column oven temperature was set at 45°C, and the run time was established as 7 min.

The mass spectrometry analysis was performed with a SCIEX 4500 triple quadrupole mass spectrometer controlled by Analyst v.1.6.3 software (SCIEX, Framingham, MA, USA). The mass spectrometry detector was operated in the electrospray ionisation mode with the data acquisition in the multiple reaction monitoring mode. The operating parameters were as follows: curtain gas (N_2_) 20, nebuliser gas (N_2_) 60, collision gas (N_2_) medium, auxiliary gas 65, ion spray voltage 4500 and temperature 450°C. The EF and CP parent → daughter ion transitions were monitored: 360 → 342/286 and 332 → 314/231, respectively. The ion transition for CP-d8 as the IS was 340 → 322. The MS/MS parameters for quantitative analysis of EF were as follows: declustering potential (DP) 100 V, collision energy (CE) for ion 1 33 V and for ion 2 48 V and collision cell exit potential (CXP) 23 V. The parameters for CP were DP 61 V, CE for ion 1 30 V and for ion 2 47 V and CXP 20 V. For CP-d8 as the IS, the following values were set, respectively for EF and CP: DP 89 and 60 V, CE 40 and 29 V and CXP 13 and 13 V.

### Validation of the analytical method

The quantitative UHPLC-MS/MS method was validated in terms of selectivity, linearity, precision (repeatability and within-laboratory reproducibility) and recovery for each matrix (haemolymph, mucus, albumen gland, muscle and hepatopancreas). The calculation of the parameters was performed according to the requirements included in the EU 2021/808 regulation (22). The limit of quantification (LOQ) of the developed method was established according to the Guidance Document EUR 28099 (56). Selectivity was determined by analysis of antibacterial-free samples spiked with EF and CP, linearity was assessed by preparing calibration curves in analysis-appropriate concentration ranges and the square of the coefficient of determination (R^2^) was determined. In terms of repeatability, concentrations of samples were determined by repeated analysis of samples (n = 6) spiked at three levels (5, 50 and 500 μg/kg or μg/L) using the same instrument and in the care of the same operator on the same day. For the within-laboratory reproducibility calculation, another two sets of fortified samples were analysed for repeatability on three different days by different operators. The coefficient of variation (CV, %) was calculated for precision. Concentrations were calculated in reference to the IS using a matrix-matched calibration curve. For the recovery, the mean measured concentrations were determined in the same experiment as repeatability. The LOQ was determined as the lowest point of the matrix calibration curve.

### Pharmacokinetic analysis

The concentration data of EF and CP in haemolymph, mucus, albumen gland, muscle and hepatopancreas were analysed using a naïve pooled population approach. The pharmacokinetic analysis was performed using PKanalix software release 3 (Simulations Plus, Research Triangle Park, NC, USA). Enrofloxacin haemolymph concentrations were analysed using a non-compartmental approach for the single-dose regimen. Maximum haemolymph (C_max_) of EF and the time required to reach it (T_max_) were acquired directly from the data. The terminal elimination half-life (HLλz) was calculated using linear least-squares regression analysis of the concentration–time curve. The area under the concentration–time curve was calculated from time zero to the last measurable concentration (AUC_0–t_) and extrapolated to infinity (AUC_0–inf_). The AUC values were estimated using the linear log trapezoidal rule for IHL administration and the linear-up log-down rule for PO administrations. Pharmacokinetic estimates were calculated if the value of AUC_rest_ % was lower than 20% of AUC_0–t_ and the square of the coefficient of determination (R^2^) of the terminal phase regression line was >0.85.

## Results

### Validation of the method

**Fig. 2.**
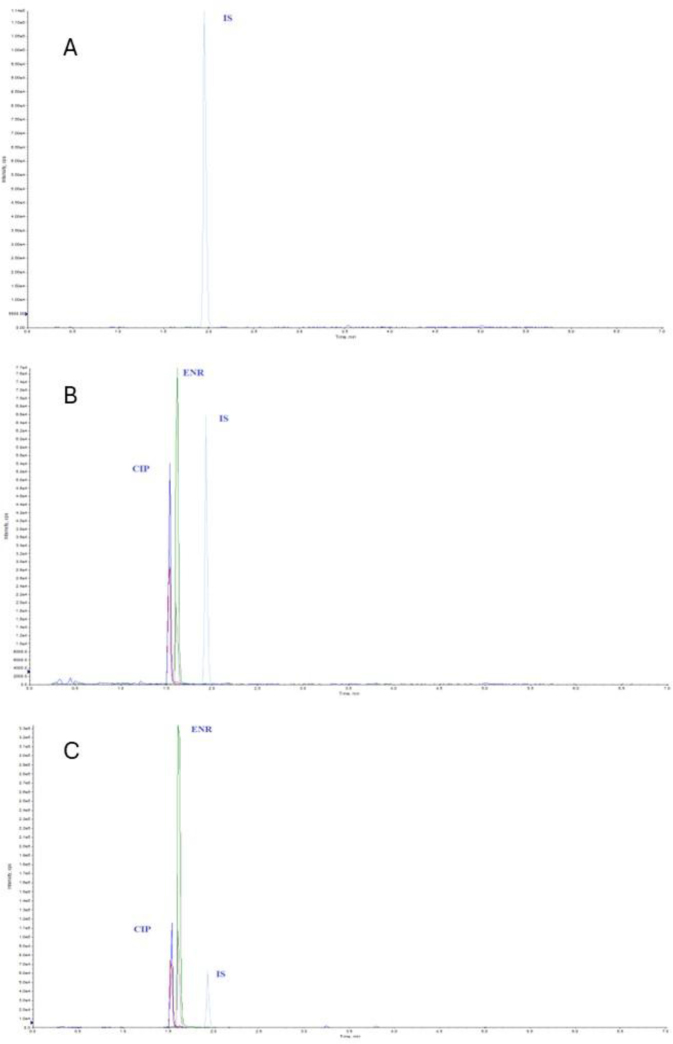
Chromatograms of year-old *Cornu aspersum maxima* muscle samples. (A) blank snail muscle sample; (B) snail muscle sample fortified with enrofloxacin and ciprofloxacin at the level of 1,000 μg/kg; (C) experimental muscle sample with enrofloxacin and ciprofloxacin 240 h after drug administration. cps – counts per second; ENR – enrofloxacin; CIP – ciprofloxacin; IS – internal standard

The analytical UHPLC-MS/MS method for the determination of EF and CP in haemolymph, mucus, albumen gland, muscle and hepatopancreas of the snails was validated in terms of selectivity, linearity, precision, recovery and LOQ. Table 1 shows the validation results of the quantitative method, presenting the parameters assessed in haemolymph, mucus and all analysed tissues. Regarding selectivity, no interfering peaks were observed in the retention times of the analytes investigated. As an example, the chromatograms of a blank muscle snail sample, a muscle sample fortified with EF and CP and an experimental muscle sample are presented in Fig. 2. The method demonstrated satisfactory linearity, repeatability, reproducibility and recovery for each matrix studied. The low LOQ values indicated the high sensitivity of the method.

**Table 1.** Validation results of the quantification analysis of enrofloxacin and ciprofloxacin in haemolymph, mucus, albumen gland, muscle and hepatopancreas of year-old *Cornu aspersum maxima* snails

Parameter	Enrofloxacin					Ciprofloxacin				
	Matrix									
	Haemolymph	Mucus	Albumen gland	Muscle	Hepatopancreas	Haemolymph	Mucus	Albumen gland	Muscle	Hepatopancreas
R^2^	0.991	0.992	0.994	0.995	0.993	0.992	0.993	0.991	0.993	0.993
Repeatability, CV,%	8.6	7.5	7.4	6.9	6.6	7.0	6.8	9.1	7.3	5.9
Reproducibility, CV, %	10.4	8.3	8.7	8.1	7.8	8.2	7.9	11.5	9.1	8.8
Recovery,%	96.6	102.8	97.5	96.1	101	94.9	105.6	99.1	95.8	99.5
LOQ (μg/L)	5	-	-	-	-	10	-	-	-	-
LOQ (μg/kg)	-	5	5	5	5	-	10	10	10	10

1 R^2^– square of the coefficient of determination; CV – coefficient of variation; LOQ – limit of quantification

### Animals

No visible adverse reactions were observed in any of the snails during or up to nine days after the drug administration. No deaths were recorded during or after the experimental sessions.

### Pharmacokinetics

**Fig. 3.**
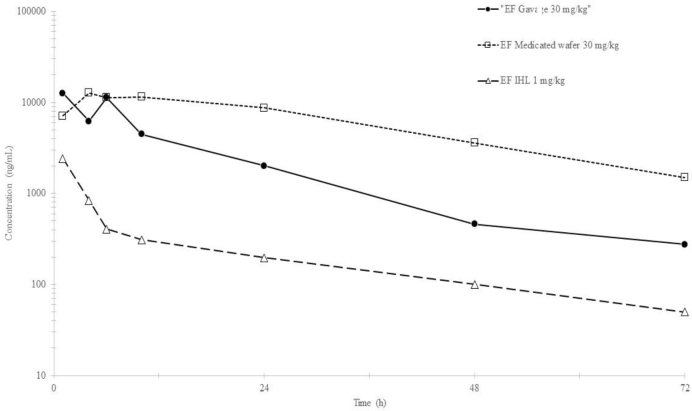
Semi logarithmic mean haemolymph concentration–time curves (± SD) of enrofloxacin (EF) following single intrahaemolymphatic (1 mg/kg), gavage and medicated wafer (30 mg/kg) administrations in year-old *Cornu aspersum maxima* snails (n = 3 × 65)

In the single-dose regimen, haemolymph concentrations of EF were still quantifiable and well above the LOQ at 72 h after both IHL and PO administration (Fig. 3). The haemolymph concentrations of CP were quantifiable up to 4 h after IHL administration at 1 mg/kg and up to 24 and 48 h after administration in the PO gavage and medicated wafer groups. These concentrations fluctuated, were not consistent with the dose, detectable only in some snails and were not amenable to pharmacokinetic analysis (Supplementary Fig. S1).

Table 2 reports the pharmacokinetic parameters of the average EF haemolymph concentrations in the three treatments. The HLλz was long (range 15 to 19 h) and the bioavailability (F%) was higher in the medicated wafer group (75%) than in the gavage group (28%). However, it was not possible to statistically analyse the data because of the study design, and these results should be considered as descriptive only.

**Fig. 4.**
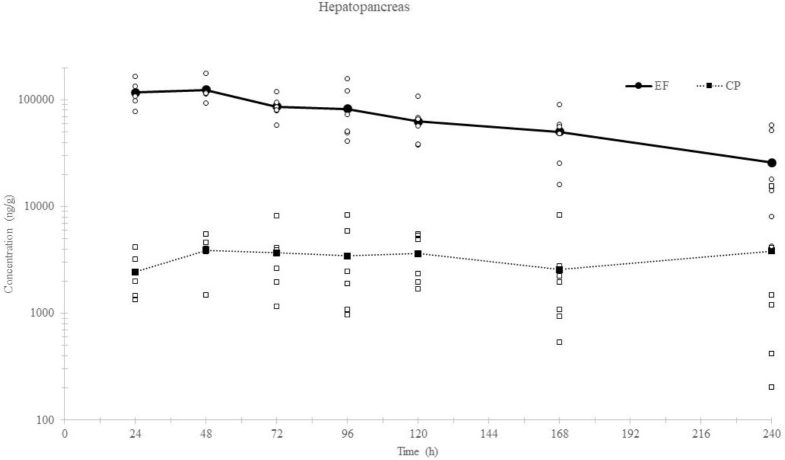
Mean hepatopancreas concentrations of enrofloxacin (EF) and ciprofloxacin (CP) following the fifth day of multiple oral (gavage) administration (10 mg/kg) to year-old *Cornu aspersum maxima* snails (n = 49). ○, □ represent the EF and CP individual sample concentrations, respectively

In the multiple-dose regimen after 5-d gavage treatment with 10 mg/kg EF, the sample type with higher concentrations of EF and CP was the hepatopancreas (Fig. 4).

**Table 2.** Pharmacokinetic parameters of enrofloxacin in year-old *Cornu aspersum maxima* snails after single intrahaemolymphatic (IHL) (1 mg/kg), gavage and medicated wafer (30 mg/kg) administrations (n = 3 × 65)

Parameter	IHL	By cannula	By wafer
R^2^	0.918	0.9585	1
λ_z_(1/h)	0.0354	0.0463	0.0366
HL λ_z_ (h)	19.5677	14.9812	18.9184
T_max_ (h)	/	0.75	4
C_max_ (ng/mL)	/	19,064	12,745
AUC_last_/D (h*ng/mL)	20,838	5,963	15,130
AUC_inf_/D (h*/ng/mL)	22,250	6,161	16,495
V_z_ (mL/kg)	1,268.755	/	/
Cl (mL*h/kg)	44.9432	/	/
MRT (h)	19.2014	15.493	30.0288

1 R^2^ – square of the coefficient of determination; λ_z_ – elimination rate constant; HLλ_z_ – terminal elimination half-life; T_max_ – time to reach peak plasma concentration; Cm_ax_ – peak plasma concentration; AUC_last_/D – area under the concentration-versus time curve calculated on the last point and corrected for the administered dose; AUC_inf_/D – area under the concentration–time curve extrapolated to infinity and corrected for the administered dose; V_z_ – volume of distribution; Cl – total body clearance; MRT – mean residence time; F% – bioavailability

The albumen gland and muscle showed similar concentrations of EF and CP, while mucus showed slightly higher concentrations (Fig. 5a, b and c). In these three latter matrices, CP was not quantifiable in all the samples. Its concentration was not quantifiable in any sample of haemolymph, while EF was quantifiable up to 240 h from the last drug administration (Fig. 6).

**Fig. 5a.**
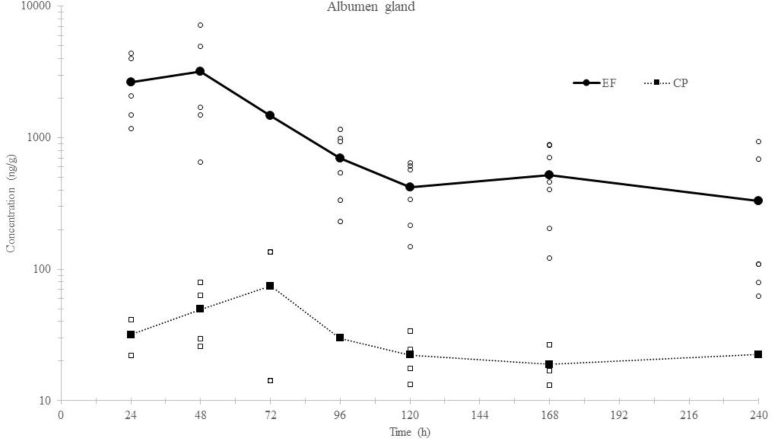
Mean albumen gland concentrations of enrofloxacin (EF) and ciprofloxacin (CP) following the fifth day of multiple oral (gavage) administration (10 mg/kg) to year-old *Cornu aspersum maxima* snails (n = 49). ○, □ represent the EF and CP individual sample concentrations, respectively

**Fig. 5b.**
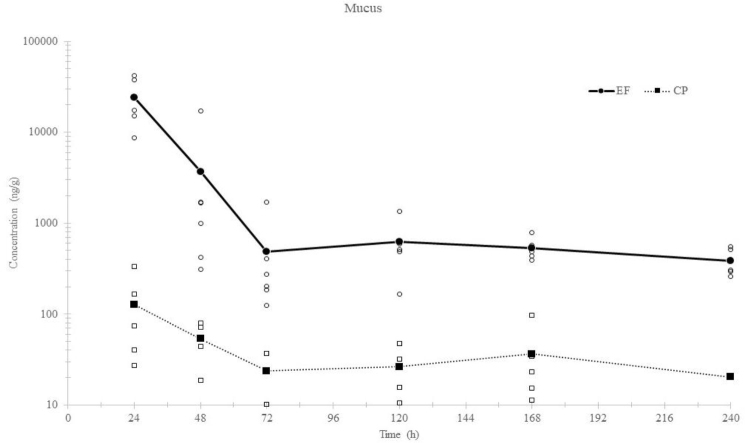
Mean mucus concentrations of enrofloxacin (EF) and ciprofloxacin (CP) following the fifth day of multiple oral (gavage) administration (10 mg/kg) to year-old *Cornu aspersum maxima* snails (n = 49). ○, □ represent the EF and CP individual sample concentrations, respectively

**Fig. 5c.**
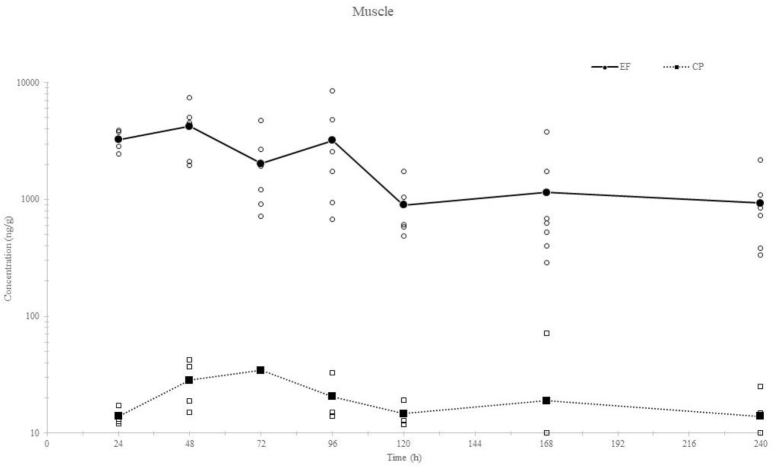
Mean muscle concentrations of enrofloxacin (EF) and ciprofloxacin (CP) following the fifth day of multiple oral (gavage) administration (10 mg/kg) to year-old *Cornu aspersum maxima* snails (n = 49). ○, □ represent the EF and CP individual sample concentrations, respectively

**Fig. 6.**
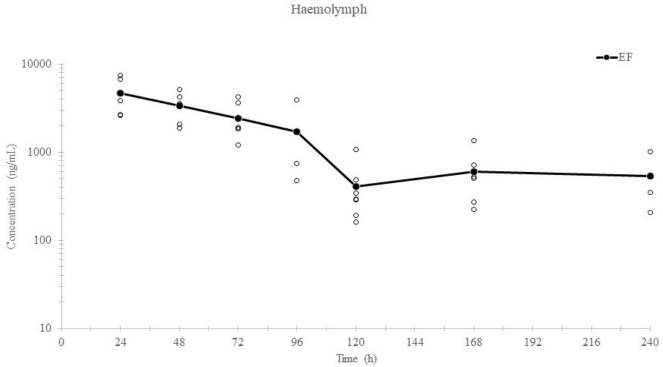
Mean haemolymph concentrations of enrofloxacin (EF) following the fifth day of multiple oral (gavage) administration (10 mg/kg) to year-old *Cornu aspersum maxima* snails (n = 49). ○ represents the EF individual sample concentration

## Discussion

The present study was designed with two aims. In the single-dose treatment regimen, the potential bioavailability and haemolymph and tissue concentrations achieved after PO administrations were evaluated. The multiple-dose treatment regimen was necessary to mimic the real-life scenario in snail breeding, to assess how EF and CP behave pharmacokinetically and to observe the potential residues of the two compounds in diverse tissues and fluids of snails. The rationales for the tissue and fluid selections were that haemolymph mirrors mammalian blood, the muscle is the main snail product eaten by consumers, the hepatopancreas may be consumed when whole young snails are served, the albumen gland is responsible for the production and storage of eggs which are consumed in the form of caviar, and mucus is a base for some cosmetic and pharmaceutical products.

As we had no information about the toxicity of EF in snails, we proceeded with caution, administering a low dose to the IHL group (1 mg/kg to avoid potential toxicity). Also lacking information about the oral bioavailability of the drug in snails, we used a higher dose in the PO groups to compensate for potential low haemolymph concentrations resulting from unpredictable low PO bioavailability. The results from the first regimen allowed a 10 mg/kg/day, 5-d multiple-dose regimen to be established, generating quantifiable concentrations with no probable toxicity to the snails. This dosage corresponds to the recommended one in most animal species (21).

The results of the haemolymph concentrations after the single dose showed a high degree of variability in all treatment groups. This may be because of the potential lack of precision in the drug’s administration, differences in subjects or a reduced animal sample size. Regarding imprecision, injection cannot be guaranteed to have been directly into the haemolymph despite the best efforts of the researchers. Highly similar animals were selected as subjects; however, no two snails were identical and differences were unavoidable. Concerning the sample size, budget and staff resources set a limit on the research, which was conceived as a preliminary study and scaled appropriately.

The first treatment regimen produced a quite long terminal elimination half-life and a variable PO bioavailability for EF. Surprisingly, the bioavailability after gavage administration (27.7 %) was only one-third of that observed after medicated wafer administration (74.6 %). This large difference was unexpected and might have been caused by issues in delivering the drug by gavage, increased absorption of the drug if administered with feed and the small group sizes. A promising result was the low concentration of CP. In many animal species, EF was de-ethylated into CP by cytochrome P450 enzymes (53, 54). Ciprofloxacin was detected in the main tissues of sheep, pigs, dogs, llamas, cows and horses, with AUC proportions of CP to EF of 20–55% (3, 11, 29, 34, 40). In many aquatic animals, the rates of transformation of EP to CP were significantly lower than those in terrestrial animals, and generally <5% of EF was converted to CP (15, 23, 24, 37, 38, 46, 58). Although in the present study the percentage of EF metabolised to CP was not assessable, it might be negligible, mimicking the metabolism reported for aquatic species as well as reptiles. In these species, it has been demonstrated that temperature plays a pivotal role in the EF : CP ratio; however, further studies are warranted to understand if this also applies to snails.

The multiple-dose regimen revealed that EF remained present in high concentrations in the snail tissues and fluids for at least 240 h after the last administration. Ciprofloxacin was also detected, but at lower concentrations; however, in haemolymph, its presence could not be confirmed, as concentrations were below the LOD. The long half-life observed after the single dose suggests that drug accumulation following five consecutive days of daily administration at 10 mg/kg may be substantial. The maximum residue limit (MRL) for EF has not been calculated for snails, but for other food-producing species it ranges from 100 to 300 μg/kg. The withdrawal time for EF in snails could be much longer than those reported for food-producing mammalian species, and be closer to those detected in some fish (6); however, this would need substantiation by comprehensive studies. Indeed, if the tissue depletion curves are inspected (Figs 4 and 5), the sum of EF and CP concentrations is above the MRL range even after 240 h from the last administration. For this reason, an experimental withdrawal period which left edible snail tissue compliant with the aforementioned MRL could not be calculated in the present study. Further studies with extended collection points are warranted.

The hepatopancreas was the tissue with the highest EF and CP concentrations, and is the site of interaction between immune and metabolic functions (5). Although the full role of this organ has not yet been established, it has been proposed to have functions that mirror those of the liver in mammals. Consistently with the distribution observed in the snail hepatopancreas, the mammalian liver has the highest EF and CP concentrations of all organs (2, 36). The results of this study indicated that the accumulation of EF in the hepatopancreas was faster and higher than in other tissues during EF administration, because of its collection role (47). This preferential distribution of EF in hepatopancreas was also consistent with the pattern of EF distribution observed in seabream (*Sparus aurata* L.), sea bass (*Dicentrarchus labrax*), turbot (*Scophthalmus maximus*), mud crab (*Scylla serrata*), Chinese shrimp (*Fenneropenaeus chinensis*) and white shrimp (*Litopenaeus vannamei*) (16, 23, 24, 25, 37, 38).

For fluoroquinolones, which operate through a concentration-time-dependent bactericidal mechanism, the ratio of the area under the curve to the minimum inhibitory concentration (MIC) is regarded as one of the most reliable indicators of efficacy (24). In the present study, however, it is premature to speculate on this surrogate because some data, such as those on plasma protein binding and the MIC of EF against pathogenic bacteria in snails, have not been assessed.

## Conclusion

To the best of the authors’ knowledge, this is the first study of the pharmacokinetics and tissue depletion of EF in snails. The production of its metabolite CP appears to be low. The two active compounds are cleared slowly. Multiple doses of 10 mg/kg administered once daily for five days appear to generate long-lasting concentrations of EF and CP in all tissues and fluids (haemolymph excluded). Further studies are warranted to confirm these preliminary results, investigate depletion over longer collection times, assess the protein binding ratio and determine MIC of EF against pathogenic bacteria in snails.

## Supplementary Material

Supplementary Material Details
