# Supplementary material for: Pharmacokinetics and residue depletion of enrofloxacin and its metabolite ciprofloxacin in land snails (Cornu aspersum maxima)
Source: J Vet Res. 2026 Apr 27;70(2):245–56. doi: 10.2478/jvetres-2026-0024 (PMC13334301; doi:10.2478/jvetres-2026-0024)
Supplement: Supplementary file 1 — Supplementary Material Details [file jvetres-2026-0024_sm.pdf]

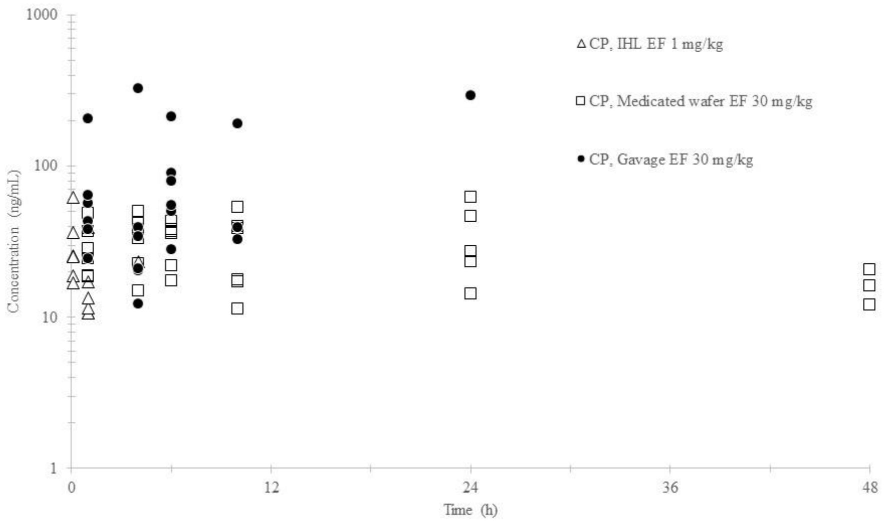

Supplementary Fig. S1. Haemolymph concentration–time curves of CP following single intrahaemolymphatic (IHL) (1 mg/kg), gavage and medicated wafer (30 mg/kg) administrations in year-old *Cornu aspersum maxima* snails (n = 3 × 65). The marks represent the individual quantifiable concentrations
